# Constitutional genetic variation at the human aromatase gene (*Cyp19*) and breast cancer risk

**DOI:** 10.1038/sj.bjc.6690071

**Published:** 1999-02

**Authors:** N Siegelmann-Danieli, K H Buetow

**Affiliations:** Division of Population Science, Fox Chase Cancer Center, Philadelphia, PA, USA

**Keywords:** breast cancer, aromatase gene, oestrogens, *Cyp19*

## Abstract

The activity of the aromatase enzyme, which converts androgens into oestrogens and has a major role in regulating oestrogen levels in the breast, is thought to be a contributing factor in the development of breast cancer. We undertook this study to assess the role of constitutional genetic variation in the human aromatase gene (*Cyp19*) in the development of this disease. Our genotyping of 348 cases with breast cancer and 145 controls (all Caucasian women) for a published tetranucleotide repeat polymorphism at intron 4 of the *Cyp19* gene revealed the presence of six common and two rare alleles. Contingency table analysis revealed a significant difference in allelic distribution between cases and controls (χ^2^ 5df = 13.52, *P* = 0.019). The allele measuring 171 bp was over-represented in cases; of 14 individuals homozygous for this allele, 13 were cases. These individuals had a higher incidence of cancer in family members and an earlier age at diagnosis than other cases. In sequencing *Cyp19*'s coding exons and regulatory regions, we discovered a perfect association between a silent polymorphism (G→A at Val80) and the high-risk genotype. Our conclusion is that constitutional genetic variation at the *Cyp19* locus is associated with the risk of developing breast cancer, with the 171-bp allele serving as the high-risk allele. © 1999 Cancer Research Campaign


*British Journal of Cancer* (1999) **79**(3/4), 456–463
© 1999 Cancer Research Campaign
Article no. bjoc.1998.0071

### Constitutional genetic variation at the human aromatase gene (*Cyp19*) and breast cancer risk

**N Siegelmann-Danieli*** **and KH Buetow**[†]

Division of Population Science, Fox Chase Cancer Center, Philadelphia, PA, USA

**Summary** The activity of the aromatase enzyme, which converts androgens into oestrogens and has a major role in regulating oestrogen levels in the breast, is thought to be a contributing factor in the development of breast cancer. We undertook this study to assess the role of constitutional genetic variation in the human aromatase gene (*Cyp19*) in the development of this disease. Our genotyping of 348 cases with breast cancer and 145 controls (all Caucasian women) for a published tetranucleotide repeat polymorphism at intron 4 of the *Cyp19* gene revealed the presence of six common and two rare alleles. Contingency table analysis revealed a significant difference in allelic distribution between cases and controls ($\chi^2$ 5df = 13.52, $P$ = 0.019). The allele measuring 171 bp was over-represented in cases; of 14 individuals homozygous for this allele, 13 were cases. These individuals had a higher incidence of cancer in family members and an earlier age at diagnosis than other cases. In sequencing *Cyp19*'s coding exons and regulatory regions, we discovered a perfect association between a silent polymorphism (G→A at Val80) and the high-risk genotype. Our conclusion is that constitutional genetic variation at the *Cyp19* locus is associated with the risk of developing breast cancer, with the 171-bp allele serving as the high-risk allele.

**Keywords:** breast cancer; aromatase gene; oestrogens; *Cyp19*

This work examined the role of constitutional genetic variation at a candidate locus, the human aromatase gene (*Cyp19*), in breast cancer development. *Cyp19* encodes for a cytochrome P450 aromatase, an enzyme catalysing the conversion of androgens into oestrogens (Tan and Muto, 1986; Mendelson et al, 1990). Aromatase activity has been demonstrated in multiple tissues including normal and transformed breast tissues (James et al, 1987; Bulun and Simpson, 1994; Santen et al, 1994; Sasano et al, 1994). Several reports suggest a positive feedback between breast tumours and aromatase activity in neighbouring breast parenchyma (James et al, 1987; Sasano et al, 1994; Schmidt and Loffler, 1994; Purohit et al, 1995). This suggests that local oestrogen production within the breast, by the aromatase enzyme, might affect breast cancer development and progression.

The *Cyp19* gene has been previously characterized and mapped to chromosome 15q21.1 (Chen et al, 1988). It is a single copy gene which spans over more than 70 kb, with the translated exons II–X spanning over only 30 kb (Harada, 1988; Harada et al, 1990; Toda et al, 1990). Multiple non-translated exons I are located at the 5′ region and control the gene expression in a tissue-specific manner and under complex hormonal regulation (Mahendroo et al, 1993; Means et al, 1991; Harada et al, 1993; Toda and Shizuta, 1993; Honda et al, 1994; Toda et al, 1995; Zhao et al, 1995) (Figure 1). These regulatory regions might also participate in the pathogenesis of malignant breast transformation because different exons I are found to control aromatase expression in normal breast (exon 1.4) and during malignant transformation (exon I.3 and promoter II) (Agarwal et al, 1996; Utsumi et al, 1996; Zhou et al, 1996*a*, *b*). The open reading frame is identical in all expressing tissues examined to date and consists of 1509 bp encoding a 503-amino-acid residue protein (Corbin et al, 1988; Harada, 1988; Means et al, 1989). Rare syndromes of complete aromatase deficiency have been described at the DNA sequence level (exon 6/intron 6 splicing mutation with an 87-bp insert described by Harada et al, 1992; a compound heterozygosity state for exon 10 mutations described by Conte et al, 1994). A common, high heterozygosity tetranucleotide simple tandem repeat polymorphism (STRP) in intron 4 has been previously described (Polymeropoulos et al, 1991). It is not known, however, whether genetic polymorphism at this locus in associated with specific phenotype.

In this study, association-based gene mapping methods were used to assess whether the *Cyp19* locus plays a role in determining breast cancer risk. Patterns of constitutional genetic variation were measured at the *Cyp19* intron for STRP (modified after Polymeropoulos et al, 1991) and contrasted in cases with breast cancer and healthy controls. To identify whether specific STRP alleles were in linkage disequilibrium with other mutations, *Cyp19* coding and regulatory regions were sequenced in cases identified with the high-risk genotype and controls with the putative low-risk genotype.

### PATIENTS AND METHODS

### Study population

Participants were consecutive non-related Caucasian women, aged 27–79 years old, living in the greater Philadelphia region and attending Fox Chase Cancer Center (FCCC) breast cancer and cancer-screening clinics and FCCC network hospitals (cancer clinics and non-cancer-related clinics), or hospital employees,

*Received 15 December 1997*
*Revised 21 April 1998*
*Accepted 11 March 1998*

*Correspondence to:* N Siegelmann-Danieli, Department of Oncology, Rambam Medical Center, PO Box 9602, Haifa 31096, Israel

Authors' current affiliations: *Department of Oncology, Rambam Medical Center, PO Box 9602, Haifa 31096, Israel; [†]Fox Chase Cancer Center, 7701 Burlhome Avenue, Philadelphia, PA 19111, USA.

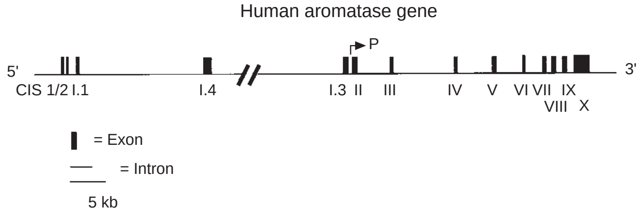

**Figure 1** *Cyp 19* coding region and major non-translated tissue-specific exon 1 in the placenta, adipose and ovarian tissues that were sequenced in cases with a high-risk genotype and controls with a low-risk genotype. Points to note: (1) coding regions: exons 2–10; (2) placenta-specific major exon 1: exon I.1; (3) regulatory elements for exon I.1: cis1/2 (Toda et al, 1995); (4) adipose-specific exon 1: exons I.4, exon I.3, promoter II (the 5′ region to exon 2,→P); [Overlapping primers were designed to include the non-translated exons and their 5′ regulatory regions (Zhao et al, 1995, 1996*a*, *b*).] exon I.4 controls aromatase expression in normal breast adipose tissue, and exon I.3 and promoter II are up-regulated during malignant transformation; and (5) ovarian-specific exon I: promoter II (→P)

from 1988 to 1994. Participation was voluntary, and participants were required to sign an institutionally-approved consent form. Cases were women diagnosed with breast cancer, controls were women with no history of cancer other than non-melanomatous skin cancer or cervical carcinoma in situ. The study group included 348 cases and 145 controls with a similar age distribution: mean age (at diagnosis for cases and at enrolment for controls) of 54.3 and 54.9 years old and median age of 53.5 and 55 years old in cases and controls respectively.

Information on tumour characteristics at time of diagnosis was abstracted retrospectively through review of the computerized medical charts. This review was blind with respect to the outcome of any genetic test used for this study. The following data were obtained: staging status according to the TNM system (American Joint Committee on Cancer, 1992); histopathological type and grade; fibrocystic changes and ductal carcinoma in situ (DCIS); oestrogen and progesterone receptor status; bilateral breast cancer; and clinical or gross-pathological multifocal tumours. These data were available for about 50–80% of participants. In 88%, staging information was based on pathological finding at time of diagnosis; in only 12% was it clinically based. The majority of cases had invasive breast tumors (96%), with 84% diagnosed with T1 and 2 (tumour up to 5 cm in greatest dimension) and 12% with T3 and 4 (tumours more than 5 cm in greatest dimension or with dermal/chest wall invasion). Axillary nodal metastases were noted in 38% of cases; in 74%, the histology type was ductal carcinoma and in 8% it was lobular carcinoma. The rest had either both or adenocarcinoma not otherwise specified. Histology grade was well to moderate in 54% and steroid receptors were positive in 63% of cases. The cases in our population had a relatively low incidence of DCIS in comparison with 1991 National Institute of Health Surveillance, Epidemiology, and End Results (SEER) data (4% vs 12.4% respectively). In other respects, the cases were comparable to women newly diagnosed with invasive breast cancer in the United States during the study period (Ries et al, 1994).

Information on family history of cancer was available for 262 cases. A positive family history of breast cancer in a first-degree family member was noted in 60 families (23%). Family history consistent with hereditary breast or breast and ovarian syndromes

**Table 1** Primers used to sequence *Cyp19* coding regions and major non-translated exons I that are transcribed in adipose, ovarian and placenta tissues (see also Figure 1)

| Exon | Primer sets | Forward oligonucleotide | Reverse oligonucleotide | Annealing temperature (°C) | Size (bp) |
|---|---|---|---|---|---|
| Cis1 | Cis1 | CCAGGAATCAGGAGACCT | TTCTAGGAGAAGCTGAGAAAGA | 56 | 303 |
| Cis2 | Cis2 | GGATTTTTTGGCACAGGAG | CCCAGTCATCATATCCCCAC | 58 | 278 |
| I.1 | ExI.1 | AGAGAGGAAGAAGAATCTGAAC | ATCTACCTGGAAAGAGTGTCTG | 59 | 310 |
| | ExI.1a | AAGGACAGGGTTCAGGGAGT | GCAGTGTTTTCCCTCTGCTC | 66 | 321 |
| | ExI.1b | AGCCTTCTGGGCTTCTCTTT | CTTCCTCTCTTTGTGCAGCA | 58 | 514 |
| I.4 | ExI.40 | TCCTGAAAGAATGTCAGCTCG | GCAGGGGTGTCAGAGTTTTC | 60 | 155 |
| | ExI.41 | AGTAGTGCATTTGAGAATGGG | AGCTGAAGACGACAGATGAA | 57 | 347 |
| | ExI.42 | TCCTTGATCCCAGGAAACAG | TGCCAAAGCACAGAACAGTC | 57 | 334 |
| | ExI.43 | AAGGAATGGTGAGAGTTTGG | TTTTTGTGTCCTGACTGTGG | 56 | 420 |
| I.3 | ExI.3 | GATTTGGCTTCAAGGGAAGA | ATCGGGTTCAGCATTTCCAA | 62 | 480 |
| 2 | Ex2 | GGACTCTAAATTGCCCCCTC | ATGATGGACCAAAATCCCAA | 57 | 246 |
| 3 | Ex3 | AGTAACACAGAACAGTTGCA | GCAATGTTAGATTTCTGGGG | 55 | 374 |
| 4 | Ex4 | AGCTGCCTCCTAGTCAAAATG | TTACAGTGAGCCAAGGTCGT | 65 | 414 |
| | Ex4a[a] | AGCTGCCTCCTAGTCAAAATG | TACCTTTCATAAAGAAGGGTCG | 58 | 238 |
| 5 | Ex5 | TCTGTAGGCTGATTCTCTG | GGTCAAGATGTGAGAGTGA | 53 | 242 |
| 6 | Ex6 | CTCAGAGCAACCTTCTTAGGC | AGAAAAGTTACCTGAGAGGCC | 56 | 288 |
| 7 | Ex7 | CATGGCAAATAAATCTGTTTCG | GGGCTATTTGGATTGGGATT | 56 | 333 |
| 8 | Ex8[b] | AGTGTCACCTCCCCTCATTT | GATATCAGATTCTTAGGAC | 52 | 324 |
| 9 | Ex9 | CCACAGGTGAGAGAGACATAA | GCTCCTTACATTCTTTGCAA | 57 | 257 |
| 10[c] | Ex10.1 | TGAATCAAACAGAGACTGAGTG | ACTCTTGGCCTCTGCTTTTTC | 57 | 538 |
| | Ex10.2 | TCTGCTCCTGTTCACACCAG | ACACTAGCAGGTGGGTTTGG | 56 | 285 |
| | Ex10.3 | TGATTAGAAAGACCAGGCCA | TCTCTTGGTTAGCCACACTAA | 56 | 489 |
| | Ex10.4 | ATTATTAGGGCCCTGTGTCT | TCTCTTGGTTAGCCACACTAAT | 59 | 480 |
| | Ex10.5 | TGAATCATTGTATGTGGTCATG | TTTCAGGGAGTTACACTGTCA | 53 | 279 |

[a]Ex4a was designed to amplify exon 4 and its immediate flanking regions, whereas primers Ex4 amplified also the intron 4 STRP region. [b]The sequencing products in both cases and controls did not include the 35-bp region located at –80 bp to –45 bp upstream to exon 8 according to gene bank data. [c]Primers Ex10.1 were used to amplify the coding region of exon 10, and Ex10.2 to 10.5 to amplify its 3′ UTR up to the second polyadenylation site.

(having two or more affected first-degree relatives with these cancers; affected first- and second-degree relatives on the maternal side of the family; or two affected second-degree relatives on paternal side of the family) was evident in 26 families (10%).

### Genetic analysis

DNA extraction from peripheral nucleated cells was performed using a salt extraction protocol (Miller et al, 1988). The STRP at intron 4 of the *Cyp19* gene was typed using primers described by Polymeropoulos et al (1991). The polymerase chain reaction (PCR) was performed on 15 ng of genomic DNA using 2.5 pmol of each *Cyp19* primer. Amplification was carried out for 35 cycles (denaturation at 94°C for 30 s, annealing at 60°C for 30 s, and extension at 72°C for 30 s). The products were denatured (5 min at 95°C) and applied to a 6% denaturing acrylamide gel along with size markers. Dried gels were exposed to radiographic films for 48–72 h.

To confirm Mendelian transmission and the allele calling for the STRP, the CYP19 locus was genotyped on the CEPH reference pedigree panel. A total of 90 independent individuals were genotyped from 45 families. A subset of 12 pedigrees was genotyped for all three generations of the families. Genetic mapping methods were carried out as described previously (Buetow, 1996).

To rigorously determine absolute allele sizes in bp, samples from 78 individuals were also genotyped using the ABI 373 fluorescent electrophoresis system. Three fluorescent deoxyuridine triphosphates (dUTP) were added to the PCR reaction described above (Prism dUTP Set, Applied Biosystems, USA), each to a different DNA sample. A lambda phage DNA digested with *Pst*I was labelled with a fourth fluorescent dye and used as internal lane standard. The products were pooled and run on a 6% denaturing acrylamide gel with an internal size standard in each lane. They were analysed using the Genotyper software to determine allele size, identification and peak height. Contingency table analysis was used to compare allele and genotype distribution between cases and controls and to assess allele distribution and tumour characteristics.

### Sequence analysis

Sequencing was performed on the ABI 377 using the ABI Prism Dye terminator Cycle Sequencing Ready Reaction Kit with AmpliTaq DNA Polymerase, FS (Applied Biosystems). Primers were designed using the Primer program (v0.5) for the regions shown in Figure 1 and are listed in Table 1. PCR products of sizes less than 300 bp were sequenced on 36-cm plates, whereas products larger than 300 bp were sequenced on 48-cm plates. The sequence generated by the ABI 377 instrument was analysed using a combination of software tools including ABI's sequence analysis software (for conversion of gel files to electropherograms), Phred/Phrap (for base determination and sequence assembly) and Conscript (for mutation/polymorphism identification within sequence assemblies). Phred and Phrap were provided courtesy of P Green, University of Washington, USA.

**Table 2** Allele frequencies in Caucasian women (expressed as percentage of a specific allele in the total group) for 692 chromosomes of cases and 284 chromosomes of controls[a]

| Allele no. | Allele size (bp) | Frequency in controls | Frequency in cases |
|---|---|---|---|
| 1 | 168 | 0.334 | 0.327 |
| 2 | 171 | 0.134 | 0.185 |
| 3 | 175 | 0.116 | 0.118 |
| 4 | 183 | 0.018 | 0.019 |
| 5 | 187 | 0.345 | 0.335 |
| 6 | 191 | 0.053 | 0.016 |

*Chi-squared analysis revealed the allele distribution to be significantly different between cases and controls at $P = 0.019$, $\chi^2$ 5df = 13.52.

### RESULTS

### Genetic mapping of the *Cyp19* locus

Genotyping was first conducted on the CEPH reference panel. The CYP19 locus was observed to be in Hardy–Weinberg equilibrium and transmitted in a Mendelian fashion. To confirm that the locus under investigation was indeed the CYP19 locus previously described in the literature, the STRP was genetically mapped using the CEPH genotype resource (Buetow, 1996). Significant pairwise linkage was observed between the CYP19 STRP and six markers localized to human chromosome 15 (D15S220, D15S117, D15S125, D15S131, D15S114 and D15S175). Complete linkage (no recombination) was observed between CYP19 and D15S220 (lod score = 16.26). Multipoint linkage analysis localized CYP19 with lod 3 support to the interval defined by loci D15S172 and D15S117 in the Cooperative Human Linkage Center's (CHLC) version 4.0 recombination–minimization map (http://WWW.CHLC.ORG). The maximum likelihood location for the locus was in the 3.5-cM interval defined by D15S648–D15S117, and is in agreement with prior mapping to 15q21.1 (Chen et al, 1988).

### Cyp19 allele's distribution in cases and controls

Cases and controls analysed by autoradiography showed six common alleles with sizes ranging from 168 bp to 191 bp (allele 1, 168 bp; allele 2, 171 bp; allele 3, 175 bp; allele 4, 183 bp; allele 5, 187 bp; allele 6, 191 bp). Two rare alleles of sizes 179 bp and 195 bp were also identified in two cases and three controls. Table 2 summarizes the frequencies of the six common alleles in the study participants (692 alleles for cases and 284 alleles for controls). The 171-bp allele (allele 2) was over-represented in cases (odds ratio 1.47, confidence interval 0.993–2.17), whereas the 191-bp allele (allele 6) was over-represented in controls. Contingency table analysis revealed the allelic distribution to be significantly different between cases and controls ($\chi^2$ 5df = 13.52, $P = 0.019$). Inclusion of the two rare alleles in the analysis did not significantly affect the results ($\chi^2$ 7df = 16.07, $P = 0.024$).

To rigorously determine absolute allele size in base pairs, we genotyped samples from 78 individuals using the ABI 373 automated electrophoresis apparatus and software. As expected, the results for the six common alleles were 100% concordant with those obtained by autoradiography. Genotyping results by both methods are shown in Figure 2 (autoradiography results from the CEPH reference panel in A, and genotyping *Cyp19* locus on the ABI 373 in B).

### *Cyp19* genotype's distribution in cases and controls: identifying a high-risk genotype

Genotype analysis revealed that homozygotes for the 171-bp allele were 5.4 times more likely to be in the cases group, with a

 © Cancer Research Campaign 1999

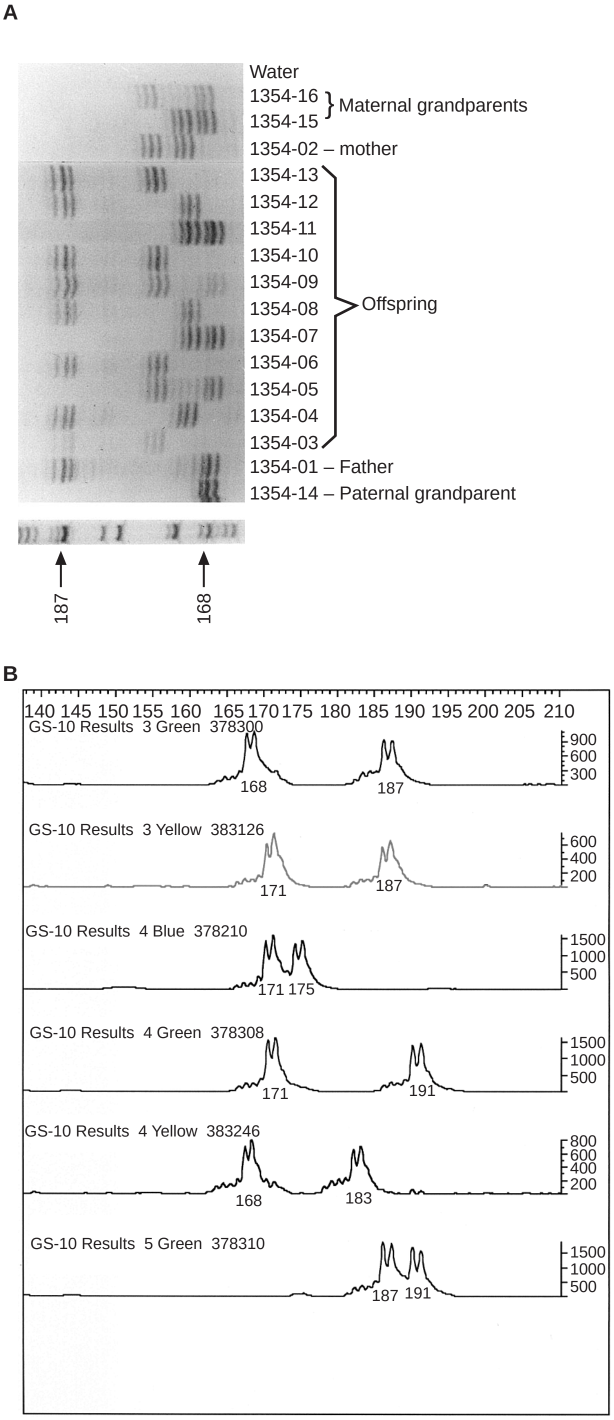

**Figure 2** Genotyping for the *Cyp19* locus. (**A**) *Cyp19* genotyping by the autoradiography method: CEPH family no. 1354. (**B**) Genotyping *Cyp19* locus on ABI 373 sequencer frequency of 3.78% and 0.70% in cases and controls respectively. Moreover, of 14 study participants homozygous for the 171-bp allele, 13 were cases. All 13 cases had unilateral invasive breast tumours, with comparable tumour characteristics to those of other cases (data not shown). They had a median age at diagnosis 5.5 years younger than other cases (mean and median age at diagnosis were 50.9 and 48 years in cases homozygous for the 171-bp allele, 54.2 and 53.5 years in total case group respectively), but this difference was not statistically significant. The only control individual homozygous for the 171-bp allele was 46 years of age at time of inclusion.

Family data were available for 13 of these individuals and are summarized in Table 3. In ten individuals, there was a first-degree family relative with a solid cancer or leukaemia. In six cases, there was a family history of breast cancer (in families 6, 9 and 10 it is suggestive of putative hereditary breast or breast/ovarian cancers). Although individuals homozygous for the 171-bp allele had a higher prevalence of a positive history of breast cancer in a first-degree family relative compared with other cases, this difference was not statistically significant ($P = 0.12$).

Two additional genotypes were disproportionally distributed among cases and controls. Heterozygotes for the 171-/187-bp alleles were 1.85 times more likely to be in the cases group (frequency of 13% and 7% in cases and controls respectively), and heterozygotes for the 187-/191-bp alleles were five times more likely to be controls (frequency of 1.12% and 5.63% in cases and controls respectively). No participants were homozygous for the 191-bp allele, which was observed to be over-represented in controls (Table 2). Other genotypes were either rare (frequency of less than 1.5%) or occurred with a similar frequency among cases and controls.

We concluded that the 171-bp allele represents a high-risk allele. Individuals homozygous for this allele are considered to carry a high-risk genotype. Heterozygotes for the 187-/191-bp alleles are considered to carry a putative low-risk genotype.

No significant association was observed between the occurrence of one or more copies of the 171-bp allele and the following cases' characteristics: menopausal status (using age 50 as a cut-off point); invasive vs non-invasive tumours; tumour size (T-stage 1 and 2 vs 3 and 4); lymph node involvement; multifocal or bilateral tumours; histology type and grade; background histology changes and receptor status.

### Sequencing results

To identify whether specific STRP allele variants were in linkage disequilibrium with other mutations at the *CYP19* gene, sequencing results were contrasted for cases with the high-risk STRP genotype (homozygous for the 171-bp allele) and controls with the low-risk STRP genotype (heterozygotes for the 187-/191-bp alleles). Sequencing efforts were targeted at the coding regions of *Cyp19* (including the 3′ UTR of exon 10 up to the second polyadenylation site), and at major non-translated regulatory exons I for adipose, ovarian and placenta tissues (see Figure 1). At least five individuals in each group were sequenced. Sequence variants confirmed by the reverse sequence were extended to additional individuals (up to 12 cases and the one control) homozygous for the 171-bp allele (DNA for one high-risk case was not available for sequencing reactions).

Table 4 summarizes the identified *Cyp19* sequence variants and their frequencies among cases with the high-risk genotype and controls with the low-risk genotype (the 12 cases and one control carrying the high-risk genotype are reported together, as they displayed the same sequence variants). Of identified sequence variants, two occurred in coding exons of the *Cyp19* gene, and were detected only among individuals carrying the high-risk genotype. The first, a silent variation at exon 3 (G→A, Val80), occurred in its homozygous state in all individuals carrying the high-risk

A control with low-risk genotype

A case with high-risk genotype

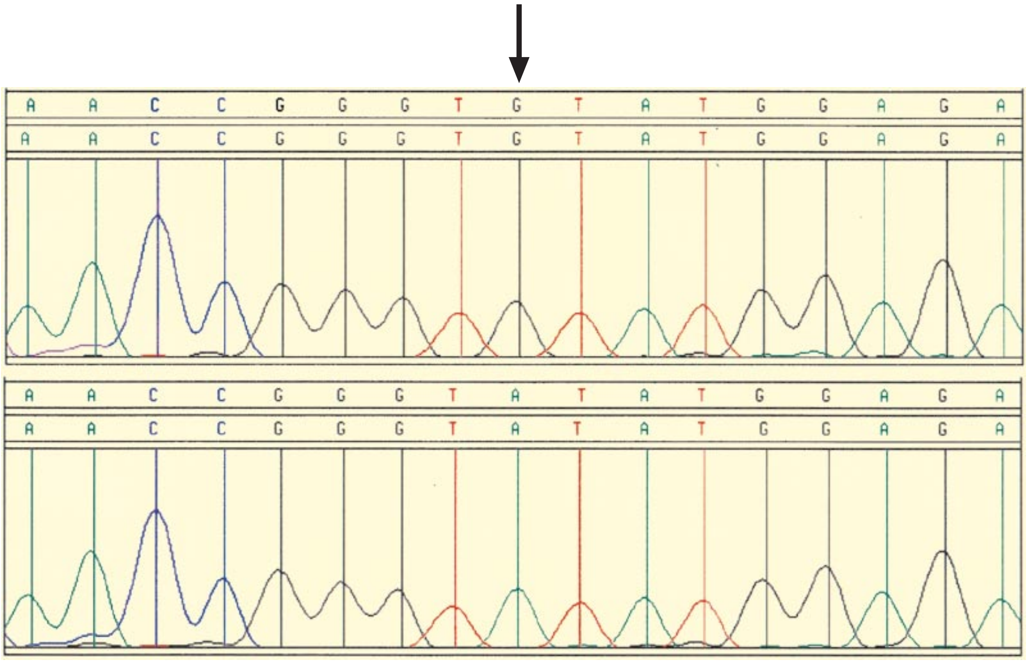

**Figure 3** G→A variation at exon 3 of the *Cyp19* gene (silent polymorphism at Val80) which appeared in complete association with the high-risk genotype

**Table 3** Family history of cancer in 14 Caucasian women homozygous for the 171-bp allele[a]

| Individual's number | Age (years) | First degree | Second or more degree |
|---|---|---|---|
| Cases | | | |
| 1 | 52 | Mother, breast cancer at 51<br>Father, lung cancer at 62 | |
| 2 | 51 | | M uncle, lung cancer<br>P uncle, lung cancer |
| 3 | 58 | Brother, lung cancer at 55 | M grandmother's brother, prostate cancer at 86 |
| 4 | 47 | Mother, breast cancer at 30 | M grandmother, leukaemia at 60<br>M grandmother's brother, cancer at 89 |
| 5 | 60 | Brother, leukaemia at 59<br>Brother, head and neck cancer | |
| 6 | 59 | Sister, ovarian cancer at 49<br>Sister, breast cancer at 50<br>Brother, lung cancer at 59<br>Brother, lung cancer at 66 | |
| 7 | 72 | Daughter, breast cancer at 49 | |
| 8 | 45 | (Not known) | (Not known) |
| 9 | 48 | | P aunt, breast cancer at 70<br>P cousin, breast cancer at 28<br>P cousin, breast cancer at 30<br>P uncle, colon cancer<br>P uncle, lung cancer<br>P uncle, CNS cancer<br>P uncle, spine cancer |
| 10 | 47 | Mother, breast cancer | M great aunt, breast cancer |
| 11 | 36 | Father, lung cancer | |
| 12 | 47 | Mother, uterine cancer | |
| 13 | 40 | | P aunt, colon cancer<br>P uncle, colon cancer |
| Control | | | |
| 14 | 46 | Brother, testicular cancer | M grandmother, uterine cancer |

[a]In seven individuals, data were available from self-administered questionnaires, and in six individuals by reviewing the medical chart with confirmation by a phone call. In individual no. 8 data were not available. M, maternal; P, paternal.

*© Cancer Research Campaign 1999*

**Table 4** Identified *Cyp19* sequence variants and their frequencies among cases with the high-risk genotype and controls with the low-risk genotype

| | Cases with the high-risk genotype (5–13 tested)[a] | Controls with the low-risk genotype (five tested) |
|---|---|---|
| Sequence variants in coding exons | | |
| Exon 3 | (13 tested) | (Five tested) |
| G→A at Val80 | AA, 1.0 | GG, 1.0 |
| Exon 7 | (Nine tested[b]) | (Five tested) |
| C→T in codon 264 (Arg→Cys264) | CC, 0.9 / CT, 0.1 | CC, 1.0 |
| Variants in non-coding regions | | |
| 5′ Region to exon I.1 | (Five tested) | (Five tested) |
| G→A at – 483 bp to exon I.1 start | GG, 0.6 / GA, 0.2 / AA, 0.2 | GG, 0.8 / GA, 0.2 |
| 5′ Region to exon I.1 | (12 tested) | (Five tested) |
| C→T at – 41 bp to exon I.1 start | CC, 0.66 / CT, 0.17 / TT, 0.17 | CC, 0.8 / CT, 0.2 |
| Intron 4 | (Five tested) | (Five tested) |
| TTTA repeats at intron 4 | Short repeat[c] | Long repeat[c] |
| Intron 5 | (12 tested) | (Five tested) |
| G→T at – 16 bp to exon 6 start | TT, 0.6 / GT, 0.4 | GT, 0.2 / GG, 0.8 |
| Intron 6 | (12 tested) | (Five tested) |
| T→A at + 36 bp to intron 6 start | AA, 0.6 / TA, 0.4 | TA, 0.2 / TT, 0.8 |
| Intron 7 | (Nine tested[d]) | (Five tested) |
| C→T at + 26 bp to intron 7 start | CC, 0.1 / CT, 0.2 / TT, 0.7 | CC, 1.0 |
| 3′ UTR | (11 tested) | (Five tested) |
| T→C at + 19 bp to intron 10 start | CC, 0.6 / TC, 0.4 | TC, 0.2 / TT, 0.8 |
| 3′ UTR | (11 tested) | (Five tested) |
| G→T at + 162 bp to intron 10 start | GG, 0.2 / GT, 0.35 / TT, 0.45 | GG, 0.8 / GT, 0.2 |
| 3′ UTR | (Five tested) | (Five tested) |
| T→C at + 1294 to intron 10 start | TT, 1.0 | TT, 0.6 / TC, 0.4 |

[a]The 12 cases and one control carrying the high-risk genotype are reported together because they displayed the same sequence variants. [b]Additionally, one CC homozygote and three CT heterozygotes were observed among high-risk cases for which sequencing results could be obtained in only one direction. [c]The sequencing results for exon 4 and its 3′ flanking region indicated that allele 2 would produce a 171-bp product and allele 5 a 187-bp product. [d]Two additional CC homozygotes and two CT heterozygotes were observed among high-risk cases for which sequencing results could be obtained in only one direction.

STRP genotype and in none of the low-risk controls (Figure 3). The second, a heterozygous state for C→T in exon 7 (Arg→Cys264), appeared in one of nine high-risk cases sequenced in both directions, and in three of four additional high-risk cases with sequencing results obtained in only one direction (frequency of 0.1–0.3 among high-risk cases). All other identified variants were in non-translated regions of the *Cyp19* gene. Of note, in concordance with the results of the automated electrophoresis genotyping analysis, the sequencing results for exon 4 and its 3′ flanking region indicated that allele 2 would produce a 171-bp product and allele 5 a 187-bp product.

The 168-bp and 187-bp alleles were the most common alleles in the study population. While the latter (together with the low-risk 191-bp allele) was found to be associated with G in Val80, we elected to check this region in individuals carrying the 168-bp STRP allele. By sequencing ten individuals homozygous for the 168-bp allele (five cases and five controls), we found all to be homozygous for G in codon Val80.

### DISCUSSION

Our study examined the association of constitutional genetic variation in the human aromatase gene (*Cyp19*) with breast cancer occurrence among Caucasian women in the greater Philadelphia region. Our cases population displayed typical features of non-selected cases with invasive breast cancer diagnosed in the United States during the study period, both in tumour characteristics and family history (see Patients and methods section). By typing close to 1000 chromosomes, we were able to identify eight alleles in the study population, sizes ranging from 168 bp to 195 bp [Polymeropoulos et al (1991) identified five alleles sized 154–178 bp, on a typing of only 46 chromosomes]. To facilitate future replicative studies, accurate allele sizes were determined by typing 78 samples using florescence-based, automated electrophoresis, and confirmed the allele's transmission in co-dominant segregation in 12 CEPH families.

Contingency table analysis revealed a statistically significant difference in allelic distribution between cases and controls. Specifically, the 171-bp allele was over-represented in cases, and the 191-bp allele was more common in controls. All but one of 14 individuals homozygous for the 171-bp allele were cases. Though not formally significant, these homozygous cases were diagnosed at a younger mean and median age than the total cases' group, and displayed a remarkably high incidence of breast and other cancers in their family histories. Preliminary data in African–American women show over-representation of individuals homozygous for

© Cancer Research Campaign 1999 

the 171-bp allele in cases with breast cancer (5 out 13 cases; one out of eight controls). These results provide additional support for the suggestion that constitutional genetic variation at or near the *Cyp19* locus is associated with the risk of developing breast cancer, with the 171-bp allele serving as the high-risk allele or at least its surrogate.

Our DNA sequencing efforts focused on the coding regions of the *CYP19* locus and the major non-translated exons I controlling its expression in adipose, ovarian and placental tissues. It included the non-translated exons I, which control aromatase expression in normal breast adipocytes (exon I.4) and during malignant breast transformation (exon I.3 and promoter II). By analysing sequences obtained for individuals with the high-risk genotypes (homozygous for the 171-bp allele) and controls with the putative low-risk genotypes (heterozygous for the 187-/191-bp alleles), we identified several common nucleotide variants. Of particular interest is the silent variation in exon 3 (G→A, Val80), which was observed to be in complete association with the high-risk genotype and was not observed in 15 sampled individuals carrying the common alleles (168-bp and 187-bp alleles) or the low-risk allele (191 bp allele). Further studies should assess its occurrence in a larger population of cases and controls, and test whether this variant can affect aromatase activity by modifying Cyp19RNA splicing or stability. This variant was previously observed by Sourdaine et al (1994) in genomic DNA from two breast tumours selected for insensitivity to the aromatase inhibitor 4-hydroxyandrostenedione (4-OHA), but not in two other breast tumours showing slightly lower basal aromatase activity and sensitivity to inhibition by 4-OHA. Sourdaine et al (1994) also observed the T→C variant at +19 to intron 10 start in the two resistant tumours (which in our work was more common among controls carrying the low-risk genotype), and a heterozygous state for the exon 7 C→T (Arg→Cys264) in only one of them. A fifth breast tumour evaluated by Sourdaine et al (1994), with no detectable aromatase activity, did not show any of the sequence variants detected in the tumours resistant to 4-OHA. Sourdaine et al (1994) identified no other coding region sequence variants in any of the breast tumours; the intron 4 STRP region and the non-translated regulatory exons I were not evaluated in their work. Taken together with our work, it is possible to theorize that the G→A variant in codon Val80, which occurred in complete association with the high-risk STRP genotype, is related to the phenotype of slightly elevated basal aromatase activity with resistance to inhibition by 4-OHA. However, only direct analysis addressing the effects of G→A (Val80) variation on aromatase expression in the breast can prove it, especially considering that Sourdaine et al (1994) selected the breast tumours in their work by their response to inhibition by 4-OHA and not their baseline aromatase activity.

Another possibility is that the intron 4 STRP itself produces an intrinsic effect on aromatase expression. Triplet repeat variations in non-coding regions of the *FMR1* gene and *MK-PK* gene are known to result in fragile X syndrome and myotonic dystrophy respectively (Fu et al, 1991; Verkerk et al, 1991; Brook et al, 1992; Mahadevan et al, 1992; Kunst and Warren, 1994). Constitutional genetic variation in variable tandem repeats located some 1000 bp downstream from the coding region of the H-*RAS*1 proto-oncogene have been shown to be associated with cancer risk in case–control studies (Krontiris et al, 1985, 1993). Functional studies have also suggested that the variable tandem repeats themselves might interact with transcription factors affecting expression of associated genes, or might alter RNA stability (Green and Krontiris, 1993; Trepicchio and Krontiris, 1993; Kennedy et al, 1995). Finally, it is possible that the high-risk STRP allele is associated with a novel breast-specific exon I not yet described. Recently, Stratakis et al (1996) reported a family with a transmitted phenotype of increased aromatase activity, which was observed to be associated with a specific STRP variant and a novel non-translated exon I. This finding implies the existence of an as-yet-unidentified variation in the 5′ flanking sequence, which controls aromatase expression and is associated with the high-risk STRP variant.

In BALB/cD2 mice, integration of the mouse mammary tumour virus to the Int5 locus (located at the 3′ UTR of mouse *Cyp19* gene) resulted in up-regulation of the aromatase gene; it was associated with the development of preneoplastic mammary tumours (Durgam and Tekmal, 1994; Tekmal and Durgam, 1995). In female Sprague–Dawley rats, treatment with the aromatase inhibitor fadrozole hydrocholoride prevented the appearance of spontaneous benign and malignant mammary tumours (Gunson et al, 1995). Understanding the biological significance of the human high-risk allele may lead to prevention of breast cancer in high-risk individuals identified by *Cyp19* genotyping.

In conclusion, our study shows a significant difference in constitutional genetic variation at intron 4 of the human *Cyp19* gene between cases and controls (Caucasian women). We identified a high-risk allele at this locus, although its biological significance has yet to be determined. Confirmatory studies are necessary to conclude that *Cyp19* genotyping can conclusively identify women at an increased risk for developing breast cancer. Ultimately, it is our hope that an understanding of the biological significance of the high-risk allele will bring us closer to a medical prevention of breast cancer, in which high-risk individuals identified with *Cyp19* genotyping are offered specific treatment affecting aromatase activity.

### ACKNOWLEDGEMENTS

This work is supported by an ASCO Young Investigator Award sponsored by Zeneca, a clinical investigator training grant CA01728 and a pilot project on a cancer genetics supplement to USPHS Institutional core grant CA06927 from the National Institutes of Health (to Dr Siegelmann-Danieli), and by an appropriation from the Commonwealth of Pennsylvania.

The authors acknowledge Katherine A, McGlynn PhD for kindly reviewing the manuscript, Michael N, Edmonson for analytical assistance and John Q Zhang and for technical assistance.

### REFERENCES

Agarwal VR, Bulun SE, Leitch M, Rohrich R and Simpson ER (1996) Use of alternative promoters to express the aromatase cytochrome P450 (CYP19) gene in breast adipose tissues of cancer-free and breast cancer patients. *J Clin Endocrinol Metab* **81**: 3843–3849

American Joint Committee on Cancer (1992) Manual for staging of cancer, 4th edn. In *Breast*, pp. 149–154. JB Lipincott: Philadelphia

Berrino F, Muti P, Micheli A, Gianfranco B, Krogh V, Sciajno R, Pisani P, Panico S and Secreto G (1996) Serum sex hormone levels after menopause and subsequent breast cancer. *J Natl Cancer Inst* **88**: 291–296

Brook JD, McCurrach ME, Harley HG, Buckler AJ, Church D, Aburatani H, Hunter K, Stanton VP, Thirion JP, Hudson T, Sohn R, Zemelman B, Snell RG, Rundle SA, Crow S, Davies J, Shelbourne P, Buxton J, Jones C, Juvonen V, Johnson K, Harper PS, Shaw DJ and Housman DE (1992) Molecular basis of myotonic dystrophy: expansion of a trinucleotide (CTG) repeat at the 3′ end of a transcript encoding a protein kinase family member. *Cell* **68**: 799–808

© Cancer Research Campaign 1999

Buetow KH (1996) Genetic mapping. In *Current Protocols in Human Genetics*. Dracopoli N, Haines JL, Korf BR, Morton CC, Seidman CE, Seidman JG, Moir DT and Smith D (eds.), section 1, pp. 1.0.1–1.10.8. John Wiley & Sons: Brooklyn, NY

Bulun SE and Simpson ER (1994) Regulation of aromatase expression in human tissues. *Breast Cancer Res Treat* **30**: 19–29

Chen S, Besman MJ, Sparkes RS, Zollman S, Klisak I, Mohandas T, Hall PF and Shively JE (1988) Human aromatase: cDNA cloning, Southern blot analysis, and assignment of the gene to chromosome 15. *DNA* **7**: 27–38

Conte FA, Grumbach MM, Ito Y, Fisher CR and Simpson ER (1994) A syndrome of female pseudohermaphrodism, hypergonadotropic hypogonadism, and multicystic ovaries associated with missense mutations in the gene encoding aromatase (P450arom). *J Clin Endrocrinol Metab* **78**: 1287–1292

Corbin CJ, Graham-Lorence S, McPhaul M, Mason Ji, Mendelson CR and Simpson ER (1988) Isolation of a full-length cDNA insert encoding human aromatase system cytochrome P-450 and its expression in nonsteroidogenic cells. *Proc Natl Acad Sci USA* **85**: 8948–8952

Durgam VR and Tekmal RR (1994) The nature and expression of *int-5*, a novel MMTV integration locus gene in carcinogen-induced mammary tumors. *Cancer Lett* **87**: 179–186

Fu Y-H, Kuhl DPA, Pizzuti A, Pieretti M, Sutcliffe JS, Richards S, Verkerk AJMH, Holden JJA, Fenwick RGJ, Warren ST, Oostra BA, Nelson DL and Caskey CT (1991) Variation of the CGG repeat at the fragile X site. Results in genetic instability: resolution of the Sherman Paradox. *Cell* **67**: 1047–1058

Green M and Krontiris TG (1993) Allelic variation of reporter gene activation by the HRAS1 minisatellite. *Genomics* **17**: 429–434

Gunson DE, Steele RE and Chau RY. (1995) Prevention of spontaneous tumours in female rats by fadrozole hydrochloride, an aromatase inhibitor. *Br J Cancer* **72**: 72–75

Harada N (1988) Cloning of a complete cDNA encoding human aromatase: immunochemical identification and sequence analysis. *Biochem Biophys Res Commun* **156**: 725–732

Harada N, Yamada K, Saito K, Kibe N, Dohmae S and Takagi Y (1990) Structural characterization of the human estrogen synthetase (aromatase) gene. *Biochem Biophys Res Commun* **166**: 365–372

Harada N, Ogawa H, Shozu M, Yamada K, Suhara K, Nishida E and Takagi Y (1992) Biochemical and molecular genetic analyses on placental aromatase (P450Arom) deficiency. *J Biol Chem* **267**: 4781–4785

Harada N, Utsumi T and Takagi Y (1993) Tissue-specific expression of the human aromatase cytochrome P-450 gene by alternative use of multiple exons 1 and promoters, and switching of tissue-specific exons 1 in carcinogenesis. *Proc Natl Acad Sci USA* **90**: 11312–11316

Honda S, Harada N and Takagi Y (1994) Novel exon 1 of the aromatase gene specific for aromatase transcripts in human brain. *Biochem Biophys Res Commun* **198**: 1153–1160

James VH, McNeill JM, Lai LC, Newton CJ, Ghilchik MW and Reed MJ (1987) Aromatase activity in normal breast and breast tumor tissues: in vivo and in vitro studies. *Steroids* **50**: 269–279

Kennedy GC, German MS and Rutter WJ (1995) The minisatellite in the diabetes susceptibility locus *IDDM2* regulates insulin transcription. *Nature Genet* **9**: 293–298

Krontiris TG, DiMartino NA, Colb M and Parkinson D (1985) Unique allelic restriction fragments of the human Ha-ras locus in leukocytes and tumor DNAs of cancer patients. *Nature* **313**: 369–374

Krontiris TG, Delvin B, Karp D, Robert NJ and Risch N (1993) An association between risk of cancer and mutations in the HRAS1 minisatellite locus. *N Engl J Med* **329**: 517–523

Kunst KB and Warren ST (1994) Cryptic and polar variation of the fragile X repeat could result in predisposing normal alleles. *Cell* **77**: 853–861

Mahadevan M, Tsilfidis C, Sabourin L, Shutler G, Amemiya C, Jansen G, Neville C, Narang M, Barcelo J, O'Hoy K, Leblond S, Earle-Macdonald J, De Jong PJ, Wieringa B and Korneluk RG (1992) Myotonic dystrophy mutation: an unstable CTTG repeat in the 3′ untranslated region of the gene. *Science* **255**: 1253–1258

Mahendroo MS, Mendelson CR and Simpson ER (*1993*) Tissue-specific and hormonally controlled alternative promoters regulate aromatas cytochrome P450 gene expression in human adipose tissue. *J Biol Chem* **268**: 19463–19470

Means GD, Mahendroo MS, Corbin CJ, Mathis JM, Powell FE, Mendelson CR and Simpson ER (1989) Structural analysis of the gene encoding human aromatase cytochrome P-450, the enzyme responsible for estrogen biosynthesis. *J Biol Chem* **264**: 19385–19391

Means GD, Kilgore MW, Mahendroo MS, Mendelson CR and Simpson ER (1991) Tissue-specific promoters regulate aromatase cytochrome P450 gene expression in human ovary and fetal tissues. *Mol Endocrinol* **5**: 2005–2013

Mendelson CR, Means GD, Mahendroo ML, Corbin CJ, Steinkampf MP, Graham-Lorence S and Simpson ER (1990) Use of molecular probes to study regulation of aromatase cytochrome P-450. *Biol Reprod* **42**: 1–10

Miller SA, Dykes DD and Polesky HF (1988) A simple salting out procedure for extracting DNA from human nucleated cells. *Nucleic Acids Res* **16**: 1215

Polymeropoulos MH, Xiao H, Rath DS and Merril CR (1991) Tetranucleotide repeat polymorphism at the human aromatase cytochrome P-450 gene (CYP19). *Nucleic Acids Res* **19**: 195

Purohit A, Ghilchik MW, Duncan L, Wang DY, Singh A, Walker MM and Reed MJ (1995) Aromatase activity and interleukin-6 production by normal and malignant breast tissues. *J Clin Endocrinol Metab* **80**: 3052–3058

Ries LAG, Miller BA, Hankey BF, Kosary CL, Harras A and Edwards BK (1994) 1973–1991 Tables and graphs, National Cancer Institute. In *SEER Cancer Statistics Review*, p. 128. Publication no. 94-2789. NIH: Bethesda, MD

Santen RJ, Martel J, Hoagland M, Naftolin F, Roa L, Harada N, Hafer L, Zaino R and Santner SJ (1994) Stromal spindle cells contain aromatase in human breast tumors. *J Clin Endocrinol Metab* **79**: 627–632

Sasano H, Nagura H, Harada N, Goukon Y and Kimura M (1994) Immunolocalization of aromatase and other steroidogenic enzymes in human breast disorders. *Hum Pathol* **25**: 530–535

Schmidt M and Loffler G (1994) The human breast cancer cell line MDA-MB231 produces an aromatase stimulating activity. *Eur J Cell Biol* **63**: 96–101

Sourdaine P, Parker MG, Telford J and Miller WR (1994) Analysis of the waromatase cytochrome P450 gene in human breast tumors. *J Mol Endocrinol* **13**: 331–337

Stratakis CA, Vottero A, Brodie A, DeArkine D, Lu O, Mitsiades CS, Meada T, Yamamoto Y, Sagara Y, Ikeda H and Shizuta Y (1996) Biochemical and molecular genetics of the syndrome of increased aromatase activity: segregation with a marker from within the P450arom gene and evidence for aberrant alternative splicing of its 5′-end mRNA. *Am J Hum Genet* **59**: A43

Tan L and Muto N (1986) Purification and reconstitution properties of human placental aromatase. A cytochrome P450-type monooxygenase. *Eur J Biochem* **156**: 243–250

Tekmal RR and Durgam VR (1995) The overexpression of *int-5/Aromatase*, a novel MMTV integration locus gene, is responsible for D2 mammary tumor cell proliferation. *Cancer Lett* **88**: 147–155

Toda K and Shizuta Y (1993) Molecular cloning of a cDNA showing alternative splicing of the 5′-untranslated sequence of mRNA for human aromatase P-450. *Eur J Biochem* **213**: 383–389

Toda K, Terashima M, Kawamoto T, Sumimoto H, Yokoyama Y, Kuribayashi I, Mitsuuchi Y, Yue W, Flor AW, Gamica A, Mitsiadis CS and Chrousos GP (1990) Structural and functional characterization of human aromatase *P-450* gene. *Eur J Biochem* **193**: 559–565

Toda K, Yang L-X and Shizuta Y (1995) Transcriptional regulation of the human aromatase cytochrome P450 gene expression in human placental cells. *J Steroid Biochem Mol Biol* **53**: 181–190

Trepicchio WL and Krontiris TG (1993) *IGH* minisatellite suppression of USF-binding-site- and Em-mediated transcriptional activation of the adenovirus major late promoter. *Nucleic Acids Res* **21**: 977–985

Verkerk AJMH, Pieretti M, Stucliffe JS, Fu Y-H, Kihl DPA, Pizzuti A, Reiner O, Richards S, Victoria MF, Zhang F, Eussen BE, van Ommen BJB, Blonden LAJ, Riggins GJ, Chastain JL, Kunst CB, Galjaard H, Caskey T, Nelson DL, Oostra BA and Warren ST (1991) Identification of a gene (*FMR-1*) containing a CGG repeat coincident with a breakpoint cluster region exhibiting length variation in fragile X syndrome. *Cell* **65**: 905–914

Utsumi T, Harada N, Maruta M and Takagi Y (1996) Presence of alternatively spliced transcripts of aromatase gene in human breast cancer. *J Clin Endocrinol Metab* **81**: 2344–2349

Zhao Y, Mendelson CR and Simpson ER (1995) Characterization of the sequences of the human CYP19 (aromatase) gene that mediate regulation by glucocorticoids in adipose stromal cells and fetal hepatocytes. *Mol Endocrinol* **9**: 340–349

Zhou C, Zhou D, Esteban J, Murai J, Siiteri PK, Wilczynski S and Chen S (1996*a*) Aromatase gene expression and its exon I usage in human breast tumors. Detection of aromatase messenger RNA by reverse transcription-polymerase chain reaction. *J Steroid Biochem Mol Biol* **49**: 163–171

Zhou D, Clarke P, Wang J and Chen S (1996*b*) Identification of a promoter that controls aromatase expression in human breast cancer and adipose stromal cells. *J Biol Chem* **25**: 15194–15202

*© Cancer Research Campaign 1999*
